# Stereotactic Body Radiotherapy for Lymph-Nodal Oligometastatic Prostate Cancer: A Multicenter Retrospective Experience

**DOI:** 10.3390/medicina59081442

**Published:** 2023-08-09

**Authors:** Francesco Cuccia, Maria Tamburo, Antonio Piras, Gianluca Mortellaro, Arianna Iudica, Antonino Daidone, Manuela Federico, Valentina Zagardo, Gianluca Ferini, Francesco Marletta, Corrado Spatola, Ivan Fazio, Sergio Filosto, Stefano Pergolizzi, Giuseppe Ferrera

**Affiliations:** 1 Radiation Oncology, ARNAS Civico Hospital, 90100 Palermo, Italy; gianluca.mortellaro@arnascivico.it (G.M.);; 2 Radiotherapy Unit, Cannizzaro Hospital, 95100 Catania, Italy; marinellatamburo@virgilio.it (M.T.);; 3 Radioterapia Oncologica, Villa Santa Teresa, 90100 Palermo, Italy; antoniopiras88@gmail.com (A.P.); antonino.daidone@villasantateresa.net (A.D.); 4 RI.MED Foundation, 90100 Palermo, Italy; 5 Department of Health Promotion, Mother and Child Care, Internal Medicine and Medical Specialties, Molecular and Clinical Medicine, University of Palermo, 90100 Palermo, Italy; 6 Radiotherapy Unit, AOU Policlinico-VE, 95100 Catania, Italy; ariannaiudicaa@gmail.com (A.I.); corrspatola@gmail.com (C.S.); 7 Radiotherapy Unit, Casa di Cura Macchiarella, 90100 Palermo, Italy; manuela.fed@gmail.com (M.F.); ivanfazio@alice.it (I.F.); 8 Radiation Oncology Unit, REM Radioterapia, 95100 Viagrande, CT, Italy; 9 Radiation Oncology Unit, La Maddalena Dipartimento Oncologico di III Livello, 90100 Palermo, Italy; sfilosto@lamaddalenanet.it; 10 Department of Radiological Science, University of Messina, 98121 Messina, Italy

**Keywords:** stereotactic radiotherapy, oligometastases, lymphnodes, prostate cancer

## Abstract

*Background:* The favorable role of SBRT for lymph-nodal oligometastases from prostate cancer has been reported by several retrospective and prospective experiences, suggesting a more indolent natural history of disease when compared to patients with bone oligometastases. This retrospective multicenter study evaluates the outcomes of a cohort of patients treated with stereotactic body radiotherapy for lymph-nodal oligometastases. *Methods:* Inclusion criteria were up to five lymph-nodal oligometastases detected either with Choline-PET or PSMA-PET in patients naïve for ADT or already ongoing with systemic therapy and at least 6 Gy per fraction for SBRT. Only patients with exclusive lymph-nodal disease were included. The primary endpoint of the study was LC; a toxicity assessment was retrospectively performed following CTCAE v4.0. *Results:* A total of 100 lymph-nodal oligometastases in 69 patients have been treated with SBRT between April 2015 and November 2022. The median age was 73 years (range, 60–85). Oligometastatic disease was mainly detected with Choline-PET in 47 cases, while the remaining were diagnosed using PSMA-PET, with most of the patients treated to a single lymph-nodal metastasis (48/69 cases), two in 14 cases, and three in the remaining cases. The median PSA prior to SBRT was 1.35 ng/mL (range, 0.3–23.7 ng/mL). Patients received SBRT with a median total dose of 35 Gy (range, 30–40 Gy) in a median number of 5 (range, 3–6) fractions. With a median follow-up of 16 months (range, 7–59 months), our LC rates were 95.8% and 86.3% at 1 and 2 years. DPFS rates were 90.4% and 53.4%, respectively, at 1 and 2 years, with nine patients developing a sequential oligometastatic disease treated with a second course of SBRT. Polymetastatic disease-free survival (PMFS) at 1 and 2 years was 98% and 96%. Six patients needed ADT after SBRT for a median time of ADT-free survival of 15 months (range, 6–22 months). The median OS was 16 months (range, 7–59) with 1- and 2-year rates of both 98%. In multivariate analysis, higher LC rates and the use of PSMA-PET were related to improved DPFS rates, and OS was significantly related to a lower incidence of distant progression. No G3 or higher adverse events were reported. *Conclusions:* In our experience, lymph-nodal SBRT for oligometastatic prostate cancer is a safe and effective option for ADT delay with no severe toxicity.

## 1. Introduction

Prostate cancer is the worldwide most frequent neoplasm in the male population [1].

For both primary disease and metastatic lesions, the role of altered fractionation is well consolidated from several sources in the literature [2,3].

The constant technological progress and the availability of imaging modalities with increased sensitivity and specificity have allowed clinicians to detect secondary lesions early in the so-called oligometastatic disease (OMD), an intermediate state of the tumor between localized and polymetastatic spread, amenable to metastases-directed approaches with proven advantages in terms of clinical outcomes [4,5].

This evidence is supported by several prospective studies also highlighting an overall survival (OS) advantage when stereotactic body radiotherapy (SBRT) is proposed in addition to standard treatments [6].

Specifically in the setting of oligometastatic prostate cancer, the role of SBRT gains attractiveness as it represents a suitable option to delay the start of androgen deprivation therapy (ADT), which may significantly affect quality of life (QoL) and increase the risk of cardiometabolic events [7].

Furthermore, SBRT might be helpful for patients already under systemic therapies, as a therapeutic option able to prolong the ongoing treatment, thus delaying the switch to the next line [8,9].

Still, the optimal combination of SBRT with ADT remains a matter of debate, with the recent recommendations of the Advanced Prostate Cancer Consensus Conference that support the use of ADT in addition to SBRT for all the oligorecurrent lesions in oligometastatic prostate cancer [10].

On the other hand, some authors hypothesize that in highly selected patients, SBRT can be used as an alternative to ADT to manage patients with a low burden of metastatic disease to postpone the start of systemic treatments or to postpone the onset of the castration-resistant status in patients with ADT already ongoing, as theorized in a currently ongoing randomized phase II trial by Zhao et al. [11].

The favorable role of SBRT for lymph-nodal oligometastases from prostate cancer has been reported by several retrospective and prospective experiences, suggesting a more indolent natural history of the disease when compared to patients with bone oligometastases. Nonetheless, also in the case of lymph-nodal oligorecurrences, the radiotherapy field remains a matter of debate, with some authors supporting the role of elective whole pelvis irradiation, as pelvic lymph nodes represent the most frequent site of oligorecurrence [12].

This retrospective multicenter study evaluates the outcomes of a cohort of patients treated with stereotactic body radiotherapy for lymph-nodal oligometastases.

## 2. Methods

This multicenter retrospective experience collects data of 69 patients treated between April 2015 and November 2022 with stereotactic body radiotherapy for lymph-nodal oligometastases. Written informed consent was acquired for all patients prior to the treatment. Inclusion criteria were as follows: up to 5 lymph-nodal oligometastases detected either with Choline-PET or PSMA-PET in patients naïve for ADT or already ongoing with systemic therapy and at least 6 Gy per fraction radiotherapy regimens for SBRT. Only patients with exclusive lymph-nodal disease were included for the purpose of the study. SBRT was proposed for all the patients with extrapelvic oligometastatic disease presentation, while for the pelvic district, in the case of oligorecurrent disease, SBRT was generally proposed in patients that received prior post-operative or curative radiotherapy extended to pelvic lymph-nodes. Within this population, three different subtypes of oligometastases were identified: oligorecurrent, oligopersistent, and oligoprogressive.

For all patients, a 3 mm thickness CT scan was acquired in treatment position, depending on the site of lymph-nodal oligometastases. Image fusion with metabolic imaging was performed to improve target volume delineation. Gross tumor volume (GTV) was considered equal to clinical target volume (CTV) and identified with the pathological lymph-node. Planning target volume (PTV) was generated with the addition of a 3–5 mm isotropic margin. Treatment planning was performed with the aim of ensuring at least 95% of the PTV was covered by 95% of the prescribed dose.

Patient positioning and setup accuracy were verified by daily image guidance with kilovoltage imaging, cone beam, or megavoltage CT scans prior to every radiotherapy session. After treatment, patients’ follow-up was scheduled every three months for the first two years, and afterwards, every six months.

The primary endpoint of the study was local control (LC), defined as the time from SBRT to the evidence of in-field failure or last follow-up visit. Secondary endpoints were considered distant progression-free survival (DPFS), i.e., the time from SBRT to the evidence of new secondary lesions or last follow-up, ADT-free survival, i.e., the time from SBRT to the activation of ADT, and overall survival (OS), i.e., the time from SBRT to the death of the patient or last follow-up visit.

Toxicity was assessed using Common Terminology Criteria for Adverse Events v4.0 (CTCAE v4.0).

For statistical analyses, baseline characteristics were reported with descriptive statistics. Survival estimates were performed using Kaplan–Meier method. Uni- and multi-variate analyses were performed to identify any potential predictive parameters for improved clinical outcomes, assuming *p* < 0.05 as statistically significant. All analyses were carried out using MedCalc v20.215 (Medcalc Software LTD, Marienkirche, Marienkirche, Belgium, BE).

## 3. Results

The present study reports the results of a multicenter retrospective experience involving six radiotherapy centers in Sicily. A total of 100 lymph-nodal oligometastases in 69 patients have been treated with stereotactic body radiotherapy between April 2015 and November 2022. The median age of the patients was 73 years (range, 60–85). At initial diagnosis, there was low-risk disease in 4 cases, intermediate risk in 36, and high risk in 29.

Only 15 patients received radiotherapy, alone or in combination with ADT as the primary treatment, while the remaining underwent radical prostatectomy as the upfront therapy with or without postoperative radiotherapy. The median disease-free interval (DFI) was 47 months (range, 12–213 months), with oligometastatic lymph-nodal disease presenting most frequently as oligorecurrent (49 cases), with oligoprogressive in 13 cases and oligopersistent in 7.

Oligometastatic disease was mainly detected with Choline PET in 47 cases, while the remaining were diagnosed by PSMA-PET, with most of the patients treated for a single lymph-nodal metastasis (51/69 cases), two in 14 cases, and three in the remaining cases.

The most frequent site of presentation of oligometastatic disease was the pelvis in 47 cases, abdomen in 16, and the thorax and other sites in 3 cases; in three patients, both pelvic and abdominal lymph-nodes were simultaneously treated.

In 32 cases, no concurrent systemic therapy was ongoing at the time of the oligometastasis diagnosis. In the remaining cases, 29 patients were on ADT, 2 on chemotherapy, and 6 with 2nd generation anti-androgens plus ADT (see Table 1). 

**Figure 1:**
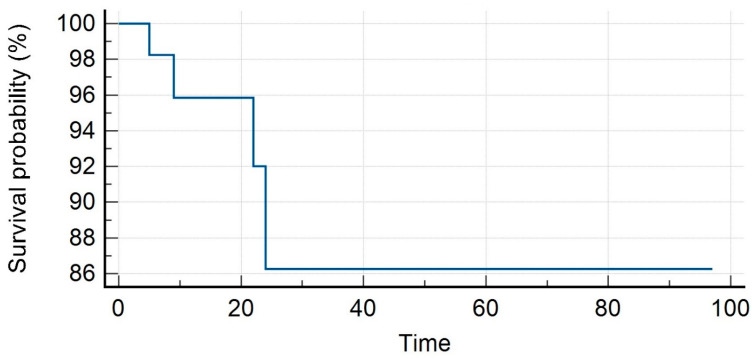
Local control.

**Figure 2:**
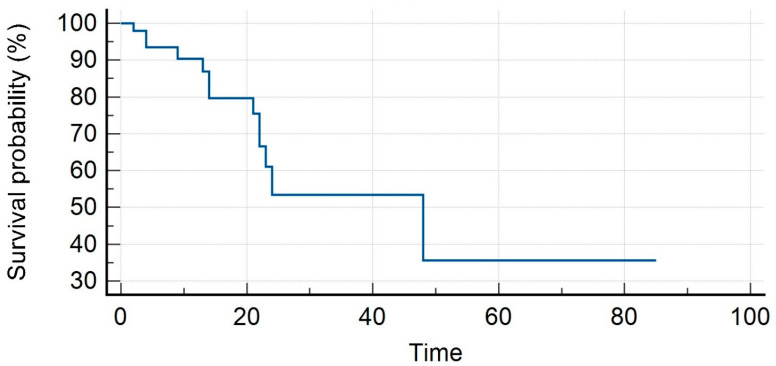
Distant progression-free survival.

**Figure 3:**
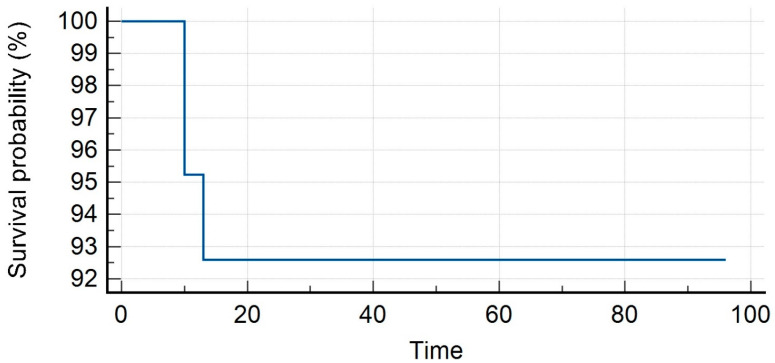
ADT-free survival.

**Figure 4:**
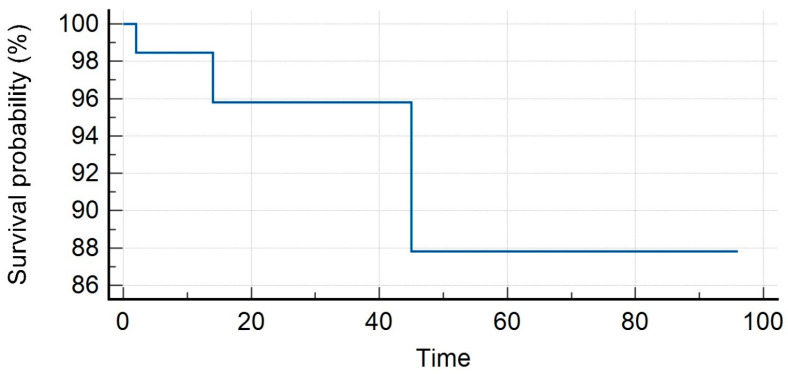
Polymetastases-free survival.

**Figure 5:**
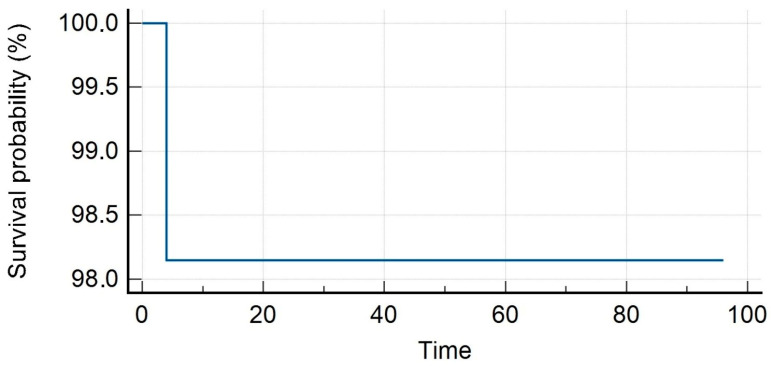
Overall survival.

The median PSA value prior to SBRT was 1.35 ng/mL (range, 0.3–23.7 ng/mL). Patients received SBRT with a median total dose of 35 Gy (range, 30–40 Gy) in a median number of 5 (range, 3–6) fractions. With a median follow-up of 16 months (range, 7–59 months), our LC rates were 95.8% and 86.3% at 1 and 2 years. Distant progression-free survival (DPFS) rates were 90.4% and 53.4%, respectively, at 1 and 2 years, with nine patients developing a sequential oligometastatic disease treated with a second course of SBRT. Polymetastatic disease-free survival (PMS) at 1 and 2 years were 98% and 96%, respectively. Six patients needed ADT after SBRT for a median time of ADT-free survival of 15 months (range, 6–22 months). The median overall survival was 16 months (range, 7–59) with 1- and 2-year rates of 98% for both (see Figure 1, Figure 2, Figure 3, Figure 4 and Figure 5).

From the univariate analysis (UA), the number of treated lesions (*p* = 0.0011) significantly related to lower rates of LC, but this was not confirmed using the multivariate analysis (MA). For DPFS, high-risk group patients were significantly associated with lower DPFS rates in the UA (*p* = 0.0006), but not confirmed in the MA, where higher LC (*p* = 0.036) and the use of PSMA-PET (*p* = 0.025) were predictors of improved DPFS rate.

Polymetastatic-free survival was found significantly related to higher pre-RT PSA values (*p* = 0.05) and lower DPFS rates (*p* = 0.0031) in the UA; in the MA, only DPFS rates kept statistical significance (*p* = 0.0091).

No factors were identified as potential predictors of outcomes in terms of ADT-free survival, likely due to the paucity of cases who received ADT after SBRT.

For OS rates, in the UA, oligopersistent lesions (*p* = 0.05) and a higher number of metastases treated with SBRT (*p* = 0.0059) were associated with worse OS outcomes, but these data were not confirmed in the MA (see Table 2). 

No acute or late G3 or higher adverse events were reported.

## 4. Discussion

SBRT in the treatment of oligometastatic disease has shown an established impact on survival outcomes [4].

Specifically for prostate cancer, the ORIOLE trial has proven superior outcomes when SBRT is proposed, especially when guided by PSMA-PET [6].

The role of new metabolic tracers has significantly changed the natural history of the tumor allowing an earlier recognition of any potential disease relapse, thus facilitating the identification of the true oligometastatic patient, with a tumor burden amenable to local treatments [13].

Especially in the case of lymph-nodal oligometastases, the optimal management remains a matter of debate for several issues. First, when the recurrence is in the pelvis, elective nodal irradiation may offer superior outcomes in terms of disease control, although at a price of a larger treatment volume with potential higher toxicity; secondly, the Advanced Prostate Cancer Consensus Conference 2019 recommended as the preferred option the combination of a systemic therapy with a local treatment of all the oligometastatic sites of the disease [10].

On the other hand, several studies in the literature support SBRT to delay the start of ADT, which is known to carry a negative impact on patients’ quality of life and a cardiometabolic toxicity profile [14,15].

All these issues need to be addressed in the perspective of a tailored approach, specifically keeping in mind that only the lymph-nodal tumor burden for prostate cancer represents a more favorable subgroup in terms of disease progression, when compared to bone or visceral oligometastases.

This multicenter experience collects the data of a cohort of patients with only lymph-nodal oligometastatic disease at the time of SBRT, including both oligorecurrent, oligoprogressive, and oligopersistent lesions, with LC as the primary endpoint.

In this series, oligometastatic disease was mainly detected with Choline-PET, with the pelvis as the most frequent site of relapse, and most patients receiving SBRT to a single lymph-node metastasis. Considering the worldwide rising availability of PSMA-PET and of the lower sensibility and specificity of Choline-PET, the outcomes in our series might be highly influenced by this potential bias, as DPFS rates were significantly influenced by the type of PET used, with improved outcomes reported in patients treated based on PSMA-PET scans.

PSMA-PET allows clinicians to detect oligometastatic disease early, also with low PSA values, with an expected improved outcome when metastasis-directed approaches are proposed [16].

This finding is confirmed in a retrospective multicenter study by Mazzola et al., in which PSMA-PET guided SBRT for both bone and lymph-nodal metastases resulted in improved ADT-free survival compared to Choline-PET guided SBRT [17].

Nonetheless, our data agree with other studies in the literature [18,19,20], including the study by Casamassima et al., in which patients who received elective nodal irradiation plus simultaneous integrated boost were also included in the analysis. Notably, this study was based only on Choline-PET imaging [21].

In our series, LC was also found to be significantly related to DPFS rates, reinforcing the assumption that metastatic foci might generate further metastases, as postulated by Gundem et al., thus supporting the role of an ablative approach on the site of disease relapse [22].

Globally, this series collects excellent LC rates at 2 years, comparable to other series reported in the literature [23,24,25,26].

PMFS-rates were found to be related to DPFS and pre-RT PSA values in the UA, but this correlation was lost in the MA, likely due to the low occurrence of polymetastatic spread in our population. Conversely, also for ADT-free survival, no statistically significant correlations were found, also because in the oligorecurrent population, very few patients needed ADT administration after SBRT. Nonetheless, our ADT-free survival rates are also comparable with other studies focusing on SBRT as a strategy to postpone ADT administration [27,28,29].

For OS, oligopersistent disease and a higher number of metastases treated were related to worse survival outcomes in the UA, but not confirmed in the MA. The limited number of deaths might be related to the more indolent natural history of the disease.

The safety profile of SBRT finds confirmation in the absence of G3 or higher adverse events, which agrees with most of the available literature, and also in the case of concurrent delivery with ADT, 2nd generation antiandrogens or chemotherapy [30].

Our study has several limitations: first of all, the retrospective nature may affect the statistical power of the data; secondly, being a multicenter experience there is a wide range of heterogeneity in treatment schedules, timing of metabolic imaging in terms of PSA thresholds, and ADT administration. Nonetheless, this study collects one of the largest series of patients with only lymph-nodal oligometastatic disease for prostate cancer, highlighting excellent outcomes in terms of disease control and toxicity.

The optimal management of lymph-nodal metastatic burden in prostate cancer remains a matter of debate, as some hypothesize a tendency to repeatedly relapse in lymph-nodal sites, thus it is still manageable with repeated courses of SBRT [31,32,33], and others supporting the need for a combination with a systemic treatment to treat potential misdiagnosed micrometastatic disease [34].

As future clinical trials like OLIGOPELVIS-2 and STORM [35,36] will provide robust evidence to address these questions, some authors also theorize a potential compromise solution, as postulated by Carrasquilla et al., combining intermittent ADT with SBRT, thus avoiding both elective nodal irradiation and long-term ADT [37].

## 5. Conclusions

In our series, SBRT for oligometastatic lymph-nodal prostate cancer results in excellent outcomes in terms of local control and disease free-survival, representing a potential therapeutic option to delay the start of ADT. Also, in terms of toxicity, SBRT appears to be safe with no major adverse events reported. Ongoing clinical trials will provide further evidence to assess the optimal combination of SBRT with systemic therapy.

## Figures and Tables

**Table 1:** Patients’ characteristics.

Characteristic	N (%) or Range
Risk group at diagnosis	Low risk = 4 (5.9%); intermediate risk = 36 (52.1%); high risk = 29 (42%)
Primary treatment	Exclusive RT = 15 (21.7%); surgery plus adjuvant RT = 22; (32%); surgery plus salvage RT = 32 (46.3%)
Disease-free interval	47 months (range, 12–213 months)
Site of lymph-nodal relapse	Pelvis = 47 (68.1%); abdomen = 16 (23.3%); thorax = 3 (4.3%); pelvis + abdomen = 3 (4.3%)
Choline PET/PSMA PET	47 (68.1%)/22 (31.9%)
Median number of metastases treated	1 lymph node = 51 (69.8%); 2 = 14 (20.2%); 3 = 7 (10.1%)
Type of oligometastases	Oligorecurrent = 49 (71.1%); oligoprogressive = 13 (18.8%); oligopersistent = 7 (10.1%)
Median PSA value prior to SBRT	1.35 ng/mL (0.3–23.7 ng/mL)
Median total RT dose	35 Gy (30–40 Gy)
Median dose per fraction	5 fx (3–6 fx)
Concurrent systemic therapy	ADT = 29 (42%); chemotherapy = 2 (3%); 2nd generation antiandrogens = 6 (8.7%)

**Table 2:** Univariate and multivariate analyses for clinical outcomes.

LC			DPFS			PMFS			OS		
	UA	MA		UA	MA		UA	MA		UA	MA
DFI	0.15	0.9	Risk Group	**0.0006**	0.84	Pre-RT PSA	0.05	0.96	Type of metastasis	0.05	0.94
N. of treated lesions	**0.0011**	0.07	Type of PET	0.17	0.025	LC	0.15	0.97	N. of treated lesions	**0.0059**	0.47
Site of lymph-node	0.16	0.4	LC	0.06	**0.036**	DPFS	**0.0031**	**0.0091**			

## Data Availability

Data available upon request.
